# Induction treatments for acute promyelocytic leukemia: a network meta-analysis

**DOI:** 10.18632/oncotarget.12451

**Published:** 2016-10-04

**Authors:** Junjie Huang, Min Sun, Zitong Wang, Qiaoxia Zhang, Jin Lou, Yun Cai, Weihong Chen, Xin Du

**Affiliations:** ^1^ Shenzhen Bone Marrow Transplantation Public Service Platform, Department of Hematology, Shenzhen Second People's Hospital, The First Affiliated Hospital of Shenzhen University, Shenzhen 518000, China; ^2^ Department of General Surgery, Taihe Hospital, Hubei University of Medicine, Shiyan 442000, China; ^3^ The University of Sydney Medical School, Sydney, NSW 2006, Australia

**Keywords:** induction treatment, acute promyelocytic leukemia, network meta-analysis

## Abstract

**Background:**

9 treatments for acute promyelocytic leukemia (APL) have been compared in many randomized controlled trials (RCT). The conclusions have been inconsistent and the purpose of this study is to conduct a network meta-analysis.

**Results:**

Rankings of event-free survival are ATRA+RIF (81.2%), ATRA+ATO (69.6%), ATO (50.6%). Rankings of complete remission are ATRA+RIF (79.3%), ATRA+ATO (64.8%), RIF (60.3%), ATO (55.9%). Rankings of avoiding differentiation syndromes are CT (84.3%), ATO (80.3%), RIF (71.6%), ATRA+RIF (49%), ATRA+ATO (40.8%).

**Methods:**

A total of 1,666 patients from 12 RCTs were enrolled. The frequentist method was used. Relative risks with 95% confidence intervals were calculated. We produced a network plot, a contribution plot, and a forest plot predictive intervals. The inconsistency factor, the surface under the cumulative ranking curve and the publication bias were evaluated.

**Conclusions:**

ATRA+ATO is eligible to be the first-line treatment for APL. ATRA+RIF is a prospective alternative to the first-line treatment. RIF or ATO should be reconsidered as another option for de novo APL.

## INTRODUCTION

Acute promyelocytic leukemia (APL) is a distinct subtype M3 of acute myeloid leukemia (AML) identified by abnormal promyelocytes and high bleeding risk [1]. Genetically, APL is characterized by a chromosomal translocation t (15, 17) and its formation of promyelocytic leukemia/retinoic acid receptor α (PML-RARα) fusion gene encodes the leukemogenic PML-RARα fusion protein [2]. The protein interferes with the maturation of myeloid cells at the promyelocytic stage, playing a central role in the pathogenesis of APL [3]. APL was the most fatal type of AML six decades ago. Nowadays, however, it has become a highly curable disease [4–6].

The first breakthrough came with the use of cytotoxic chemotherapy (CT) [7]. It helps patients achieve complete remission (CR) yet provided a low overall survival rate due to severe complications [8]. The introduction of differentiation therapy with all-trans retinoic acid (ATRA) rendered remission more easily, but around 30% of patients would relapse and were often resistant to further treatment with ATRA [9–11]. A subsequent combination of ATRA with chemotherapy raised the event-free survival (EFS) rate [12–14]. In the 1990s, arsenic trioxide (ATO) was initially used to treat relapsed APL patients. Then it was proven to be effective in de novo APL as well [15–17]. Notably, ATRA and ATO target the RARα and PML moieties of the fusion protein differently [18, 19]. A combination of these two drugs was observed to have significant improvements in the prognosis of APL by several studies [20–22]. To overcome the limitation that ATO must be intravenously administered during hospitalization, an orally active formulation of arsenic named tetra-arsenic tetra-sulfide (AS4S4) was engineered [23]. Another As4S4-containing formula, the Realgar-Indigo naturalis formula (RIF), was developed by several groups in China [24, 25]. Recently, a pilot study shows that the combination of RIF and ATRA is effective, convenient, and economical [26]. In order to compare the efficacy and safety of the different induction treatments for APL, a number of randomized controlled trials (RCTs) were conducted [27–47]. However, the conclusions of these trials have not been completely consistent due to different sample sizes, group characteristics, and clinical settings.

A systematic review and quantitative synthesis of data from different RCTs can be achieved by a meta-analysis. Although several meta-analyses have been published, there are limitations in these traditional meta-analyses [48–50]. They can only combine results from homogeneous studies researching the exact same treatment comparisons. Furthermore, it is not feasible to compare more than two treatments simultaneously. However, a network meta-analysis is able to compare three or more treatments by using a common comparator. In addition, it can synthesize the results of direct and indirect comparisons simultaneously to obtain a more accurate and precise statistical result. Therefore, we aim to perform a network meta-analysis to evaluate different induction treatments for APL.

## RESULTS

### Study selection, characteristics and assessment

**Figure 1:**
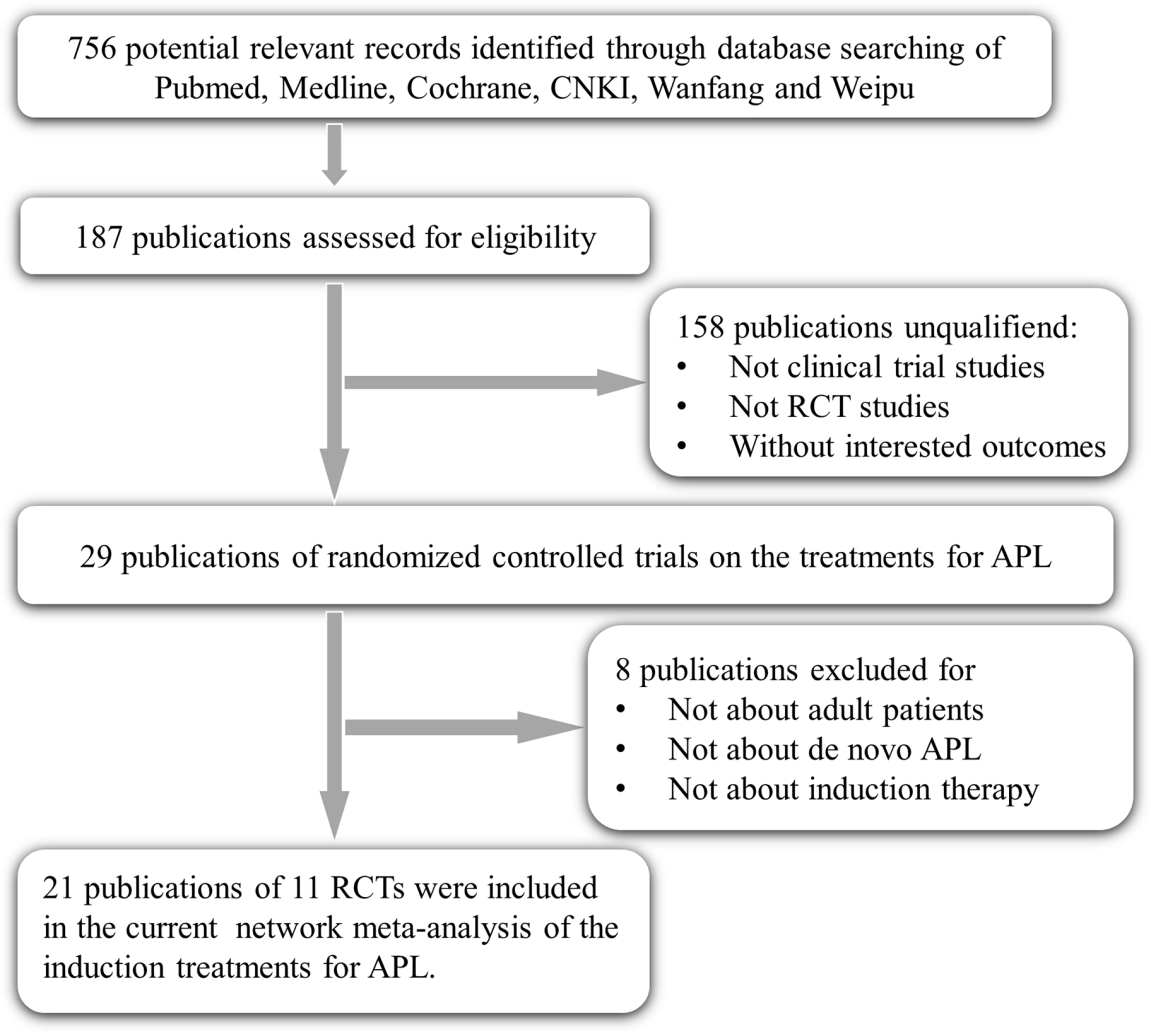
Flow chart of RCT selection

As presented in Figure 1, a total of 187 records were initially identified as eligible. 158 irrelevant studies were sorted out as they were non-clinical-trial studies, non-RCT studies, or without interested outcomes. 8 more articles were also excluded by a complete read of the articles, for that they were studies of children, relapsed APL, consolidation or maintenance therapy. Lastly, 21 publications reporting 11 RCTs with a total sample size of 1666 patients were included in this network meta-analysis. Table 1 presents these trials were carried out during 1991 to 2013 in China (6), Europe (4) and the USA (1). Four single-agent treatments of CT, ATRA, ATO and RIF were reported in 2, 6, 3 and 1 studies respectively. Four double-agent treatments of ARTA+CT (4), ATO+CT (1), ATRA+ATO (4) and ATRA+RIF (1) were also studied. Only one study reported on triple-agent treatment of ATRA+ATO+CT.

**Table 1:** Characteristics of 11 RCTs enrolled in the network meta-analysis

Trial Name	Trial Year	Country	Author	Comparison	Sample	Age	Male%	WBC (×10^9^/L)
*APL 91 trial*	1991–1993	France	Fenaux *et al.*	ATRA vs. CT	101	40(6–67)	52%	2.5
*INT0129*	1992–1995	America	Tallman *et al.*	ATRA vs. CT	346	38(1–81)	52%	2.1
*APL 93 trial*	1993–1996	France	Fenaux *et al.*	ATRA vs. ATRA+CT	208	43(2–64)	50%	1.3
*APL RJ 96*	1996–1998	China	Niu *et al.*	ATO vs. ATO+CT	11	41(24–60)	27%	2.2
*APL SX 96*	1996–1998	China	Zhang *et al.*	ATRA vs. ATO	75	39(12–62)	59%	4.3
*APL HB 99*	1999–2002	China	Ren *et al.*	ATRA+CT vs. ATRA+ATO+CT	95	34(14–68)	56%	8.1
*APL RJ 2001*	2001–2003	China	Shen *et al.*	ATRA vs. ATO vs. ATRA+ATO	61	39(14–74)	54%	7.3
*RIF Phase II*	2005–2006	China	Qian *et al.*	RIF vs. ATRA	147	37(23–54)	48%	12.7
*GIMEMA*	2007–2010	Italy	Lo-Coco *et al.*	ATRA+CT vs. ATRA+ATO	156	44(19–70)	49%	1.5
*RIF Phase III*	2007–2011	China	Zhu *et al.*	ATRA+RIF vs. ATRA+ATO	231	38(15–60)	55%	2.1
*AML17*	2009–2013	UK	Burnnett *et al.*	ATRA+ATO vs. ATRA+CT	235	47(16–77)	51%	2.6

**Figure 2:**
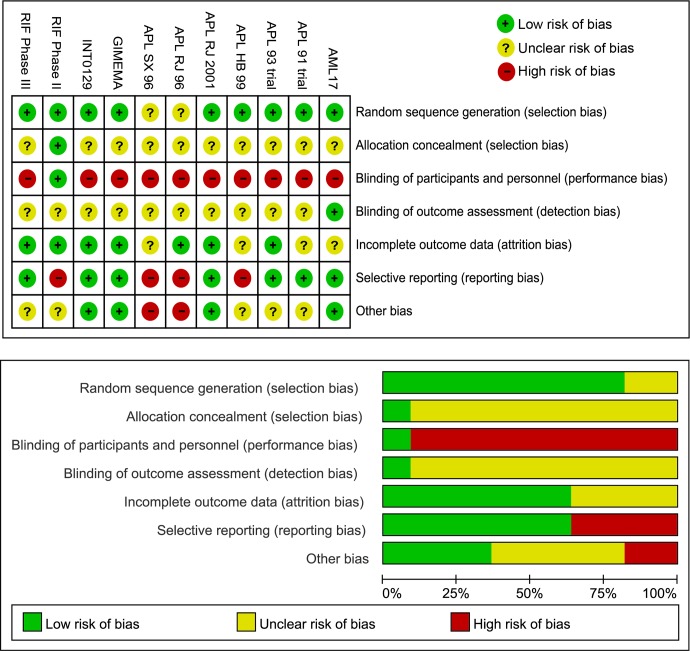
Risk of bias of the included RCTs

There are four RCTs with sample sizes of less than 100 and seven RCTs with sample sizes of more than 100. The features of most RCTs in age, gender and WBC count are not significantly different except for two studies. One study reported only 11 patients and 3 of them were males. Another study included patients with a median WBC count of more than 12.7 × 10^9^/L. The risk of RCT bias is demonstrated in Figure 2. These RCTs are well-designed in the domain of random sequence generation as only two studies are regarded as to have unclear risks of bias. Most of them did not mention about adequate allocation concealment. As for blinding of participants and personnel, only one study adopted and described the method of blinding. Other studies were open label, which may have resulted in performance bias. In terms of blinding of the outcome assessment domain, nearly all studies are rated as to have an unclear risk of bias. Eight RCTs have a low risk of bias in the domain of incomplete outcome data. Four studies have high risks of bias in terms of selectively reporting results. Two studies have high risks of bias in other biases domain.

### Network evidence, contribution and inconsistency

**Figure 3:**
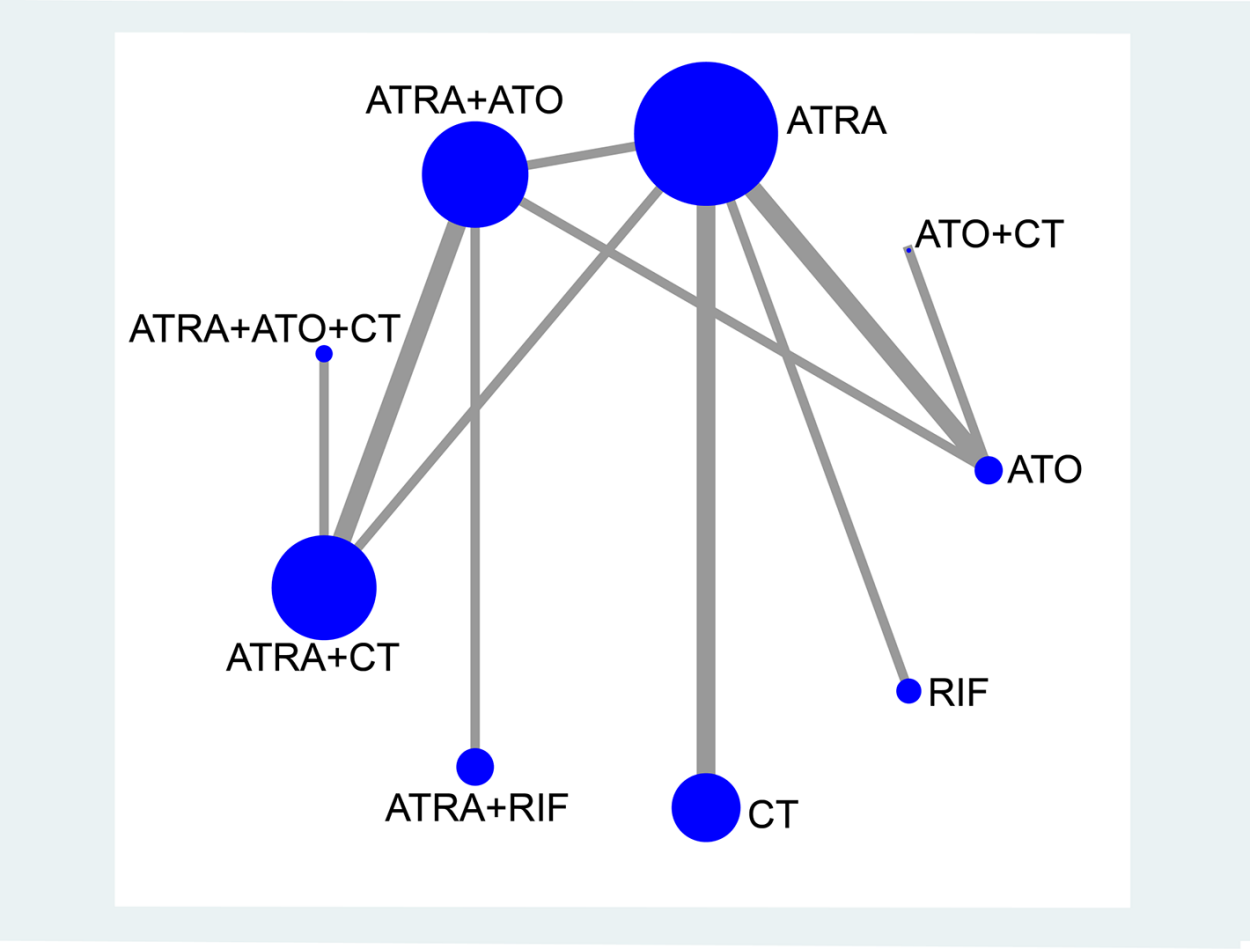
Network plot of treatment comparisons The size of each node represents the total sample size of treatment. The thickness of each line represents the total number of RCTs that compare each other. Abbreviation: CT, chemotherapy; ATRA, all-trans retinoic acid; ATO, arsenic trioxide; RIF, realgar-Indigo naturalis formula.

**Figure 4:**
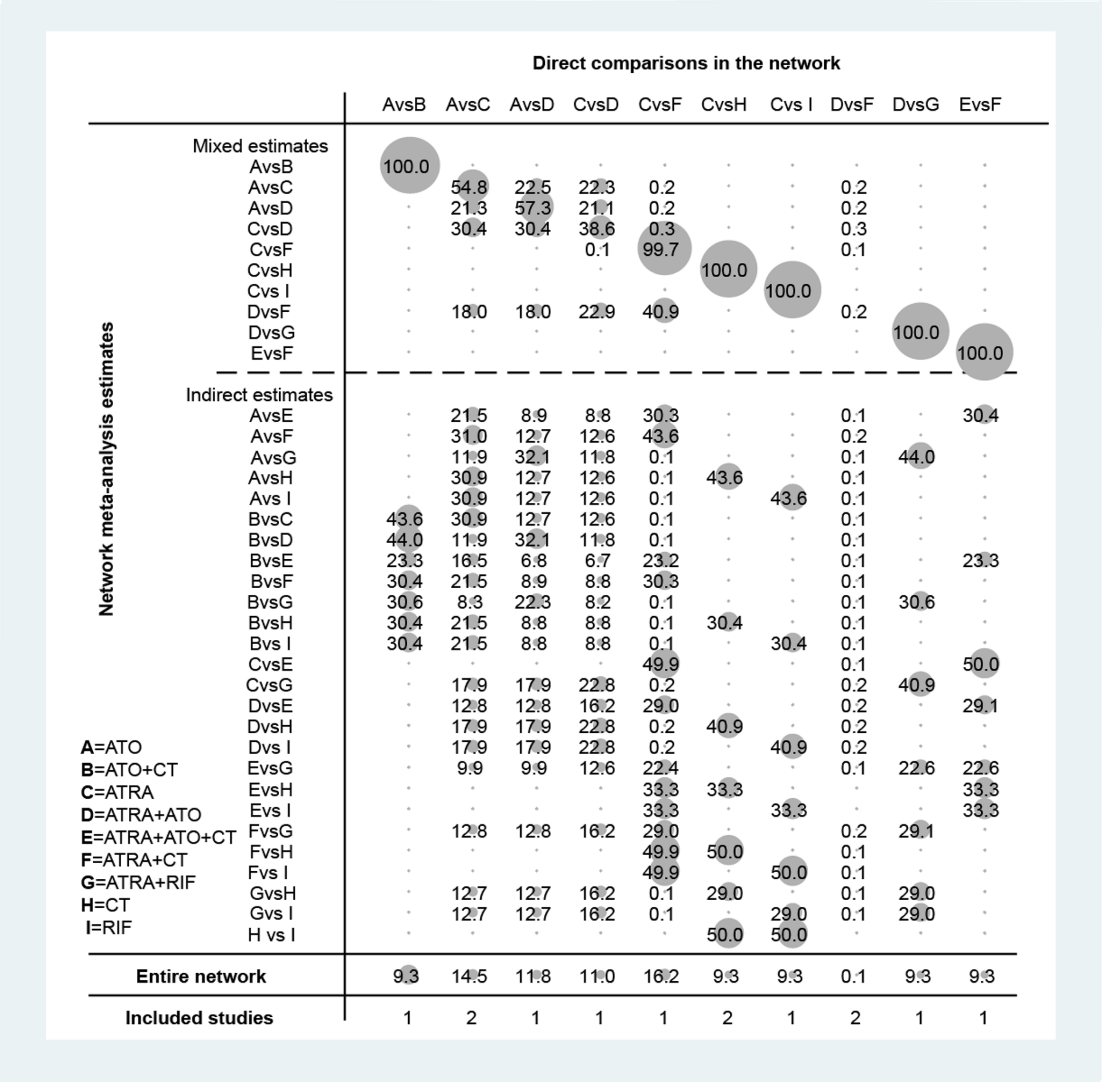
Contribution plot of the included RCTs The columns refer to the direct comparisons and the rows refer to all possible pairwise comparisons. Abbreviation: A, ATO; B, ATO+CT; C, ATRA; D, ATRA+ATO; E, ATRA+ATO+CT; F, ATRA+CT; G, ATRA+RIF; H, CT; I, RIF.

The evidence-based network is presented in Figure 3. This analysis includes 9 induction treatments for APL, namely CT, ATRA, ATO, RIF, ATRA+CT, ATO+CT, ATRA+ATO, RIF+ATRA, and ATRA+ATO+CT. It can be seen that ATRA, ATRA+ATO and ATRA+CT are the most studied treatments in RCTs, while few RCTs studied RIF, ATO+CT and ATRA+ATO+CT. Notably, although there was only one comparison of CT, its sample size and number of RCTs was relatively large. As for ATO, despite the fact that it was included in three comparisons, its sample size was small. Figure 4 shows the contribution plot for every direct comparison. Among these, 5 comparisons are informed by direct evidence alone (ATO vs. ATO+CT, ATRA vs. CT, ATRA vs. RIF, ATRA+ATO vs. ATRA+RIF, ATRA+ATO+CT vs. ATRA+CT), 5 comparisons are by mixed evidence (ATO vs. ATRA, ATO vs. ATRA+ATO, ATRA vs. ATRA+ATO, ATRA vs. ATRA+CT, ATRA+ATO vs. ATRA+CT), and 26 comparisons are by indirect evidence alone.

In terms of the overall contribution of the network, ATRA vs. ATRA+CT (16.2%) and ATO vs. ATRA (14.5%) have the most influential evidence. ATRA+ATO vs. ATRA+CT (0.1%) has the least informative direct evidence. As illustrated in Table 2, this network consists of two triangular loops, including (ATO)-(ATRA)-(ATRA+CT) and (ATRA)-(ATRA+ATO)-(ATRA+CT). The 95% CI of *IF* values was truncated at zero at the endpoints of CR (*RoR* = 1.05, 1.10; *P* = 0.78, 0.36), EFS (*RoR* = 1.38, 1.29; *P* = 0.42, 0.16) and DS (*RoR* = 1.14, 1,22; *P* = 0.95, 0.84), indicating there is no evidence of significant inconsistency. However, significant heterogeneity was observed in the endpoints of ED (*IF* = 0.88; *RoR* = 2.41), RT (*IF* = 14.5,*RoR* = 1.98E+06) and HT (*IF* = 1.07, 0.89; *RoR* = 2.92, 2.45).

**Table 2:** Results of inconsistency in the network meta-analysis

Endpoints	Loop	*IF*(95%CI)	*RoR*(95%CI)	*Z* test	*P* z
CR	(ATO)-(ATRA)-(ATRA+CT)	0.05 (0.00, 0.39)	1.05 (1.00, 1.47)	0.278	0.78
CR	(ATRA)-(ATRA+ATO)-(ATRA+CT)	0.09 (0.00, 0.28)	1.10 (1.00, 1.33)	0.915	0.36
ED	(ATRA)-(ATRA+ATO)-(ATRA+CT)	0.88 (0.00, 3.91)	2.41 (1.00, 50.0)	0.752	0.45
RT	(ATO)-(ATRA)-(ATRA+CT)	14.5 (13.0, 16.0)	1.98E+06	19.49	0.00
EFS	(ATO)-(ATRA)-(ATRA+CT)	0.32 (0.00, 1.10)	1.38 (1.00, 3.01)	0.802	0.42
EFS	(ATRA)-(ATRA+ATO)-(ATRA+CT)	0.25 (0.00, 0.61)	1.29 (1.00, 1.84)	1.391	0.16
HT	(ATO)-(ATRA)-(ATRA+CT)	1.07 (0.00, 4.51)	2.92 (1.00, 91.3)	0.610	0.54
HT	(ATRA)-(ATRA+ATO)-(ATRA+CT)	0.89 (0.00, 3.38)	2.45 (1.00, 29.2)	0.706	0.48
DS	(ATO)-(ATRA)-(ATRA+CT)	0.13 (0.00, 4.57)	1.14 (1.00, 96.8)	0.059	0.95
DS	(ATRA)-(ATRA+ATO)-(ATRA+CT)	0.19 (0.00, 2.04)	1.22 (1.00, 7.65)	0.208	0.84

Abbreviations: CR, complete remission; ED, early death; RT, remission time; EFS, event free survival; HT, hepatic toxicity; DS, differentiation syndrome; IF, inconsistency factor; RoR, ratio of two odds ratios; CI, confidence interval.

### Network comparisons, ranks and bias

**Figure 5:**
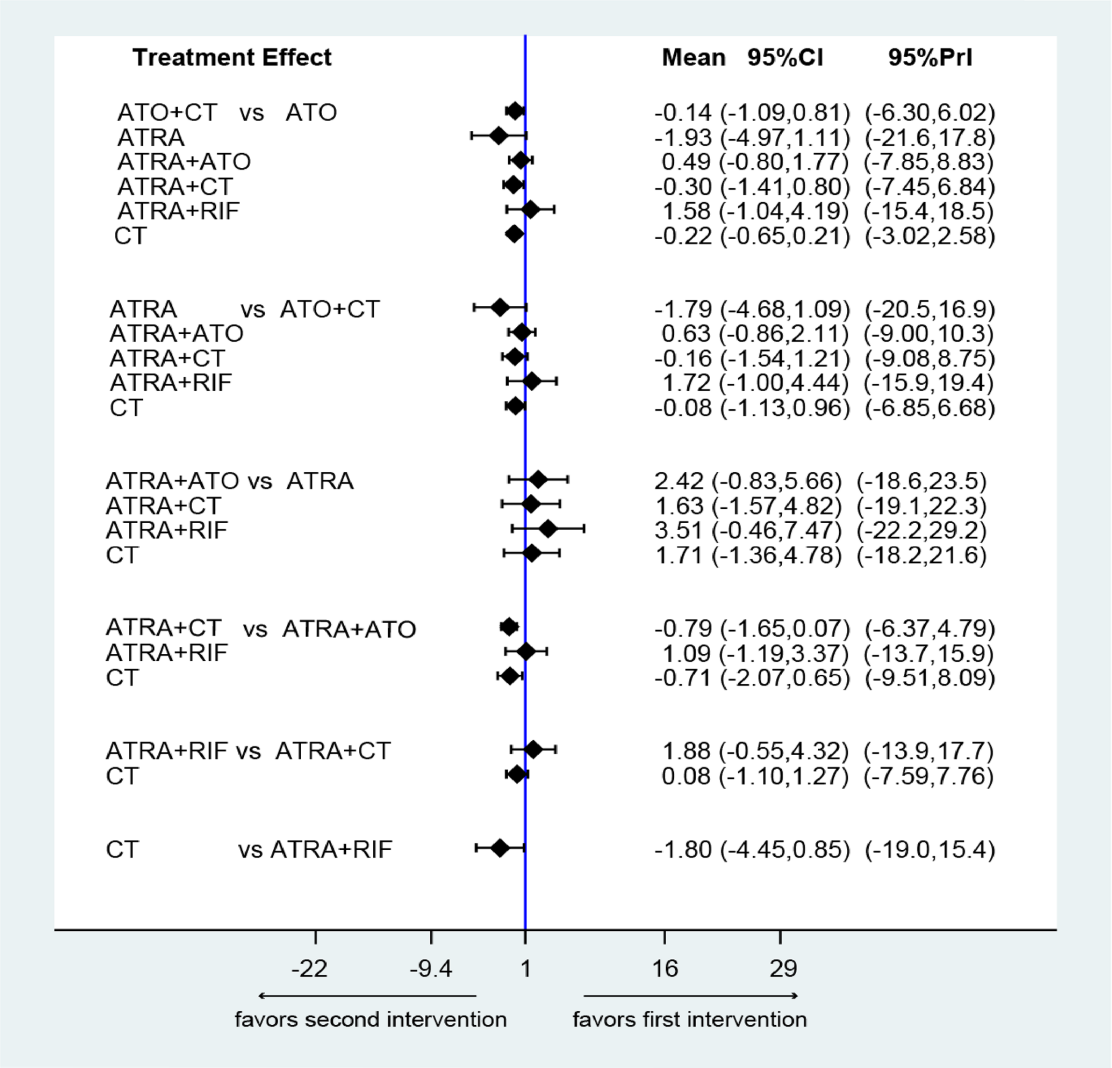
Confidence interval plot for the network analysis on a logarithmic scale The black solid lines represent the confidence interval for summary odds ratios for each comparison. The blue line is the line of no effect (odds ratio equal to 1).

**Figure 6:**
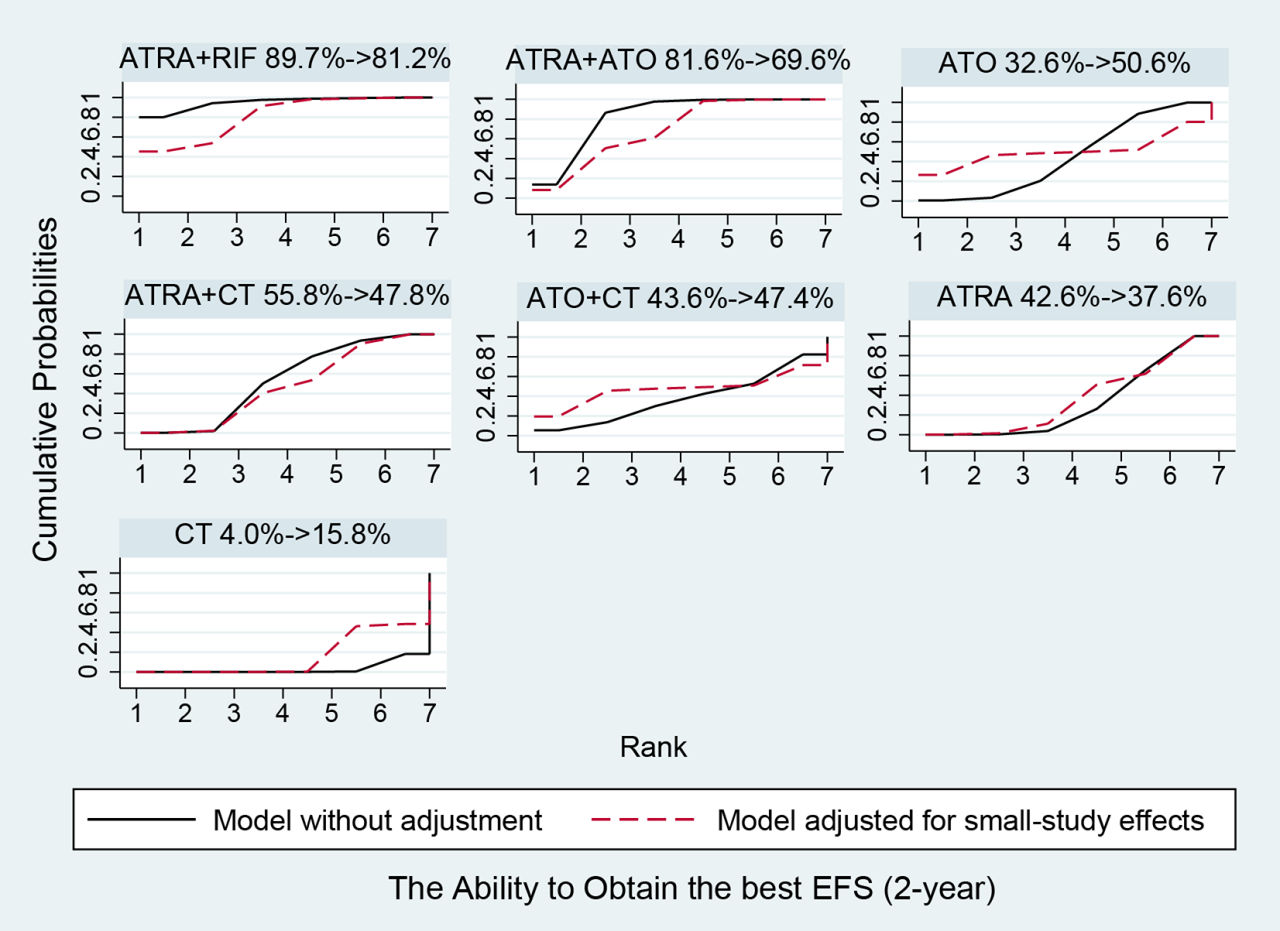
Surface under the cumulative ranking curves for the treatments in event free survival Black solid lines correspond to the unadjusted model and red dashed lines to the adjusted for small effects model.

**Figure 7:**
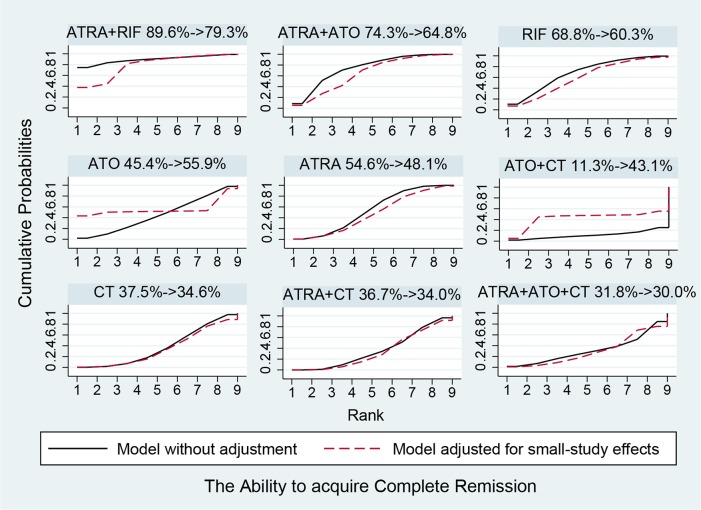
Surface under the cumulative ranking curves for the treatments in complete remission Black solid lines correspond to the unadjusted model and red dashed lines to the adjusted for small effects model.

**Figure 8:**
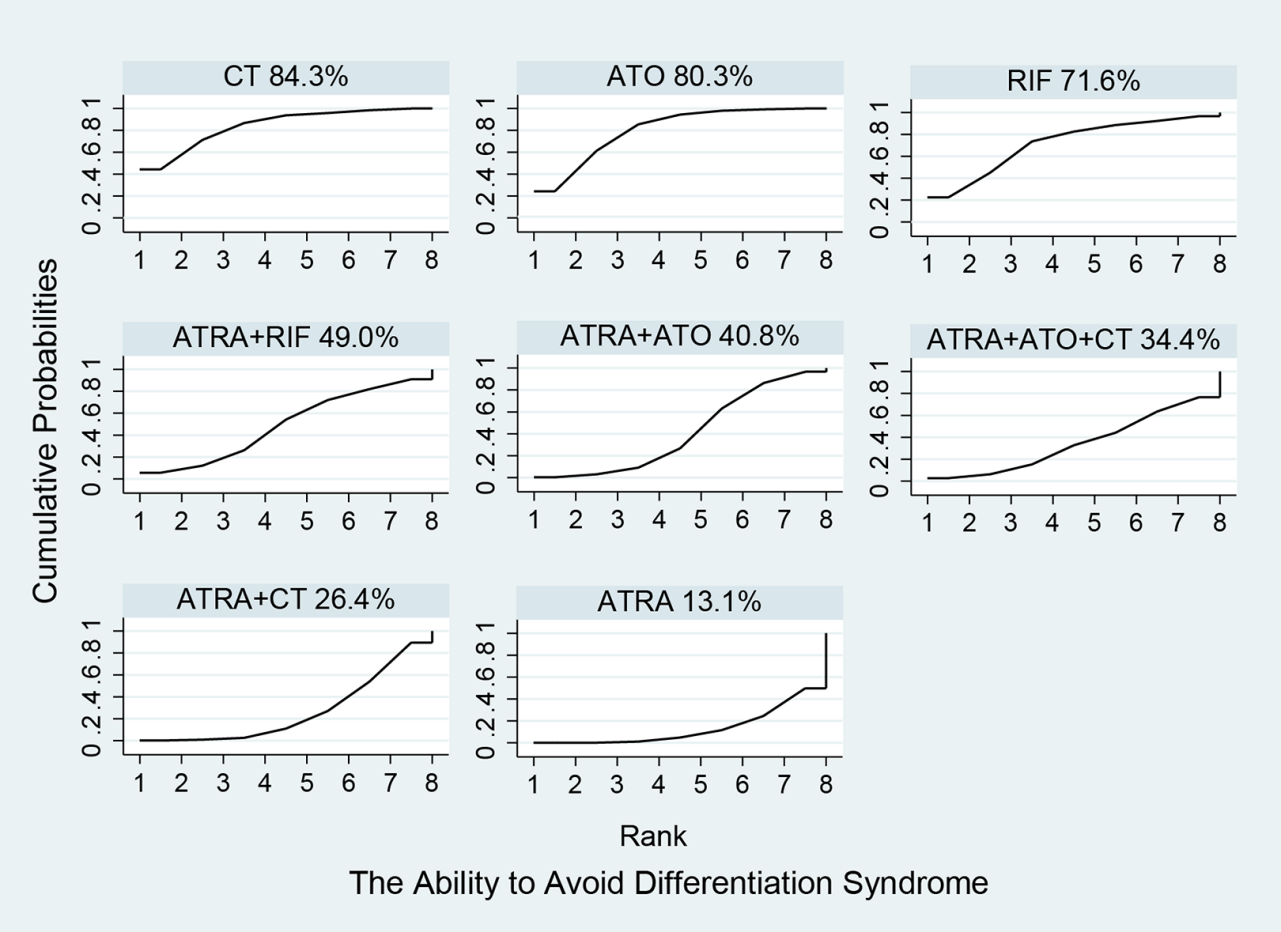
Surface under the cumulative ranking curves for the treatments in differentiation syndrome

Figure 5 shows the estimated summary effects with 95% CI for all comparisons. Although the confidence intervals and predictive intervals suggest that more RCTs are required for more statistically significant results, the forest plot gives us the impression that the treatments of ATRA+RIF, ATRA+ATO and ATO are more favorable than the other treatments. The induction treatment relative ranking of estimated cumulative probabilities of APL is demonstrated in Figures 6–8. The SUCRA value rankings of EFS are ATRA+RIF (81.2%), ATRA+ATO (69.6%), ATO (50.6%), ATRA+CT (47.8%), ATO+CT (47.4%), ATRA (37.6%), and CT (15.8%). The SUCRA value rankings of CR are ATRA+RIF (79.3%), ATRA+ATO (64.8%), RIF (60.3%), ATO (55.9%), ATRA (48.1%), ATO+CT (43.1%), ATRA+CT (36.7%), CT (34.6%), and ATRA+ATO+CT (30.0%). The SUCRA value rankings of avoiding DS are CT (84.3%), ATO (80.3%), RIF (71.6%), ATRA+RIF (49%), ATRA+ATO (40.8%), ATRA+CT (26.4%), and ATRA (13.1%).

**Figure 9:**
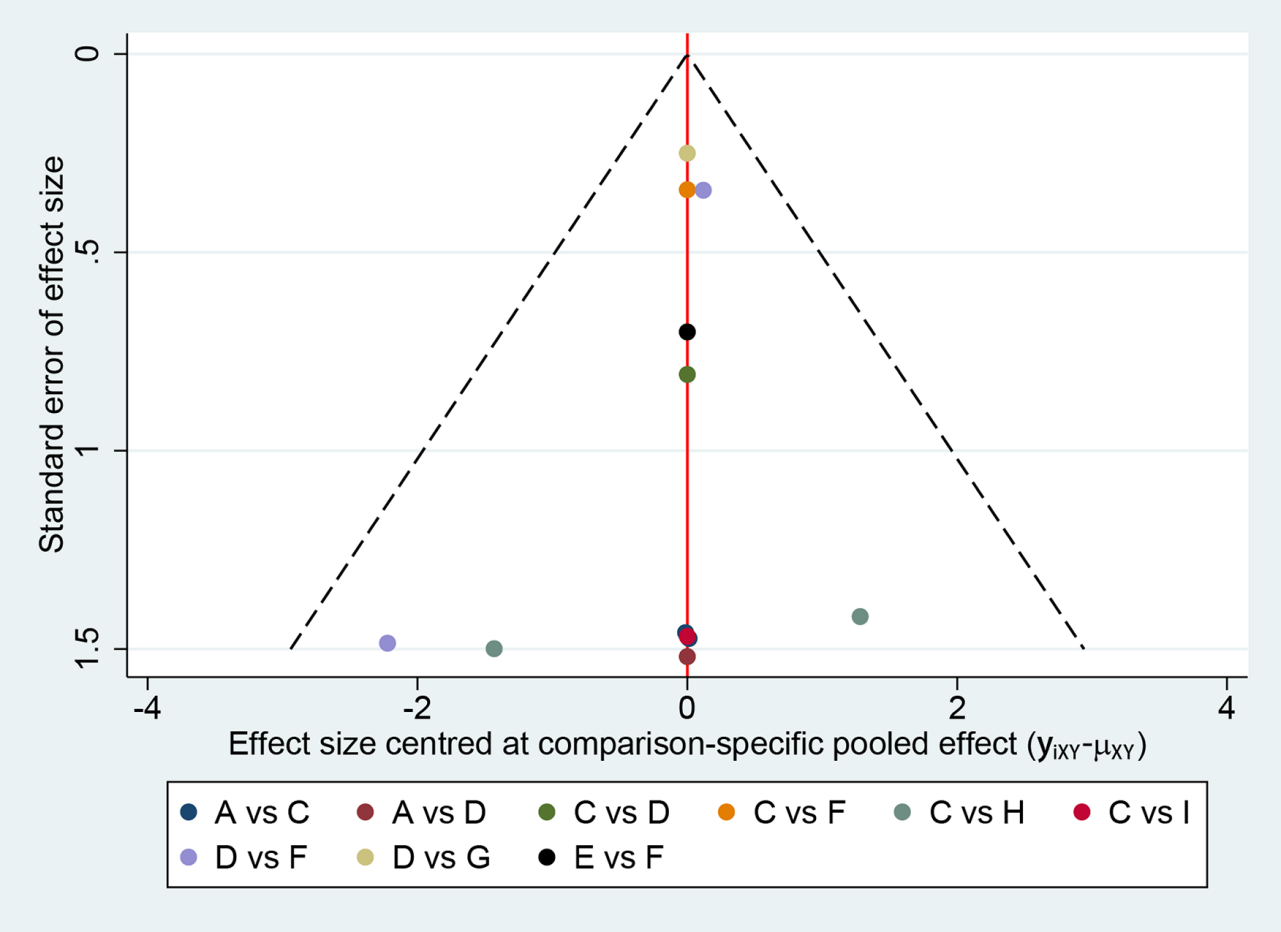
Comparison-adjusted funnel plot for the network meta-analysis The red line suggests the null hypothesis that the study-specific effect sizes do not differ from the respective comparison-specific pooled effect estimates. Different colors represent different comparisons.

Figure 9 presents the funnel plot for the network. All the included studies symmetrically distribute around the vertical line (*x* = 0), indicating no significant publication bias in this network analysis.

## DISCUSSION

We adopted different methods to prevent potential bias. RCT methodological quality assessment shows the APL SX 96 (ATRA vs. ATO) trial may have a high risk of bias. However, the evidence network proves that it is not the only study compares ATRA with ATO, reducing the risk of bias. The contribution plot demonstrates direct comparisons do not influence the entire network significantly (all are below 20%), including one study with high-risk APL patients (9.3%). There was none with statistically significant inconsistency in all included endpoints (*P* > 0.05) except for RT (*P* = 0.00). With further evaluation by *RoR*, HT and ED showed high inconsistency (*RoR* larger than 2). RT, HT, and ED were therefore excluded in the analysis to ensure the reliability. Adjusted models of SUCRA were adopted to avoid potential bias caused by the small study of APL RJ 96. As no data of DS was obtained in the APL RJ 96, there is no need for an adjusted model to be built. Lastly, the symmetrically distributed funnel plot indicates low risk of publication bias.

In Figures 6–8, the SUCRA values provide the hierarchy for the nine active treatments. ATRA+RIF was observed with the highest ranking in EFS and CR with SUCRA values of 81.2% and 79.3%, respectively. This is consistent with a recent RCT conducted by Zhu et al. [45]. In 2014, the National Comprehensive Cancer Network adopted ATRA+ATO as first-line treatment for APL [51]. Our results also found that ATRA+ATO can obtain good outcomes in EFS (69.6%) and CR (64.8%). In the single-agent induction treatments for APL, using RIF or ATO ranks first in EFS and CR compared to using ARTA or CT alone, which also conforms to the previous evidence [52–54]. The analysis also concludes that treatments with additional therapy of CT bring no benefit to EFS and CR. Additionally, treatments without ATRA could avoid DS much better than the ATRA-containing treatments.

Collectively, based on the published materials, RCTs ATRA+ATO is eligible to be first-line treatment for APL. Although ATRA+RIF shows a promising future to be an alternative to the current first-line treatment, more RCTs are required to confirm this suggestion. Another unique finding of our study is that a single agent of RIF or ATO could possibly be reconsidered as another non-inferior option for de novo APL, based on the following meta-analysis results: i) RIF or ATO is proved to be ranked highest among single-agent induction treatments for APL in EFS and CR. ii) There are much less events of differentiation syndrome in the treatment of RIF or ATO. iii) The combination of ATRA with RIF or ATO will increase the adverse events and medical cost, reducing the life quality of patients. However, the sample size of RIF or ATO treatment enrolled in the RCTs is extremely small (with a total of 73 and 27, respectively). More RCTs comparing RIF or ATO to first-line treatment are needed to explore the possibility.

In conclusion, the network meta-analysis indicates that ATRA+ATO is eligible to be first-line treatment for APL. ATRA+RIF is a prospective alternative to the ATRA+ATO. RIF or ATO should be reconsidered as an option for de novo APL. More well-designed RCTs are required to confirm these findings.

## MATERIALS AND METHODS

### Search strategy and eligibility criteria

We searched PubMed, MEDLINE, the Cochrane Central Register of Controlled Trials, EMBASE, China National Knowledge Infrastructure, Wanfang and Weipu Databases for all RCT studies that investigated the treatments of up to February 2016. The following search terms were applied: (“acute promyelocytic leukemia” OR “APL” OR “M3”) and (“treatment” “therapy” OR “medicine” OR “chemotherapy” OR “CT” OR “all-trans retinoic acid” OR “ATRA” OR “arsenic trioxide” OR “ATO” OR “Realgar-Indigo naturalis formula” OR “RIF”) and (“randomized controlled trials” OR “RCT”) without language restriction. The studies enrolled in the current network meta-analysis meet the following criteria: patients with newly diagnosed adults of APL; interventions with standard dose and duration of CT, ATRA, ATO or RIF in the induction therapy; comparisons of age, gender, white blood cells count, and follow-up of more than 2 years; outcomes of complete remission (CR), early death (ED), remission time (RT), event free survival (EFS), hepatic toxicity (HT) and differentiation syndrome (DS); study design is randomized controlled trial. The exclusion criteria were: duplicated publications; studies with insufficient data; with big difference in characteristics between groups; retrospective studies; low quality clinical trials.

### Data extraction and quality assessment

The relevant data were extracted from the articles by two investigators. The following information was obtained: name, date, place, report author, and country or study group of the clinical trials; baseline characteristics including age, gender, ethnicity, interventions across the groups; results of CR rate, ED event, RT, EFS rate, and adverse event including HT and DS. The Cochrane Collaboration's tool was adopted to assess the risk of bias in these randomized trials [55]. The risk of bias covers 7 domains, including random sequence generation, allocation concealment, blinding of participants and personnel, blinding of outcome assessment, incomplete outcome data, selective reporting, and other bias. Any disagreement was discussed with a third investigator until a consensus was reached.

### Statistical analysis

The network meta-analysis was performed by STATA 14.0 (Stata Corp, College Station, TX). 2-year EFS rate was defined as the primary endpoint. CR rate, ED event, RT, HT and DS were defined as the secondary endpoints. Relative risk (RR) with 95% confidence intervals (CIs) was calculated using the random-effects model or fixed-effects model for investigating treatment effects [56, 57]. Z test was conducted to assess the significance of overall effect size. A *P value* of less than 0.05 was considered statistically significant.

A network plot was produced to represent the overall information of the trials included in the analysis. Nodes size represents the number of trials for each treatment and lines thickness represents the number of available direct comparisons [58]. The contribution of each direct comparison to each network estimate was calculated according to the variance of the direct treatment effect and the network structure, later summarized in a contribution plot [59].

After constructing a heterogeneity matrix, the frequentist method was applied to the fitted meta-regression model. The model covariates as the basic parameters and assumed that heterogeneity is independent of the comparison between effect sizes from multi-arm studies [60]. Inconsistency refers to the differences between direct and various indirect effects estimated for the same comparison. We investigated possible sources of inconsistency using inconsistency factor (*IF*) among studies in each closed loop. If the 95% CIs of *IF* values are truncated at zero or the *P value* of z-test is higher than 0.05, it indicates that there is no statistically significant inconsistency [61]. Note that IF is the logarithm of the ratio of two odds ratios (*RoR*) from direct and indirect evidence in the loop. *RoR* values truncate at one would also indicate consistency [62]. A forest plot of the estimated summary effects, along with confidence intervals and corresponding predictive intervals (PrI) for all comparisons, summarizes the relative mean effects, the impact of heterogeneity and predictions on each comparison in one plot [63].

We estimated the probability of a treatment being ranked at a specific place according to the outcome using SUCRA (surface under the cumulative ranking curve). SUCRA is a simple transformation of the mean rank, providing a hierarchy of the treatments and accounts for the location and the variance of all relative treatment effects. The higher the SUCRA value is, the higher possible ranking of the treatment is. SUCRA was adjusted by a model of network meta-regression accounting for small-study effects, using the variance of the log-odds ratios as covariation [64].

The publication bias was evaluated by a ‘comparison-adjusted’ funnel plots whose horizontal axis presents the difference between study-specific effect sizes and the corresponding comparison-specific summary effect. The funnel plot should be symmetrical near the zero line if there is no publication bias [65].
